# Effect of acute and long-term exercise on leptin levels in depressed outpatients

**DOI:** 10.1186/s12889-023-17362-4

**Published:** 2023-12-14

**Authors:** Darlene Heinen, Andreas Heissel, Stephan Heinzel, Thomas Fydrich, Andreas Ströhle, Michael A. Rapp, Heike Vogel

**Affiliations:** 1 Social- and Preventive Medicine, Department of Exercise and Health Sciences, University of Potsdam, Potsdam, Germany; 2 Research Group Molecular and Clinical Life Science of Metabolic Diseases, Faculty of Health Sciences Brandenburg, University of Potsdam, Potsdam, Germany; 3 Social- and Preventive Medicine, Department of Sports and Health Sciences, Intra-Faculty Unit Cognitive Sciences, Faculty of Human Science, and Faculty of Health Sciences Brandenburg, Research Area Services Research and E-Health, University of Potsdam, Potsdam, Germany; 4 Sport-Gesundheitspark Berlin E.V./Zentrum Für Sportmedizin, Berlin, Germany; 5 Clinical Psychology and Psychotherapy, Department of Education and Psychology, Freie Universität Berlin, Berlin, Germany; 6 Institute of Psychology, Department of Educational Sciences and Psychology, TU Dortmund University, Dortmund, Deutschland; 7 Department of Psychiatry and Psychotherapy, Campus Charité Mitte, Charité - Universitätsmedizin Berlin, Berlin, Germany; 8 Department of Psychology, Humboldt University Berlin, Berlin, Germany; 9 Research Group Genetics of Obesity, German Institute of Human Nutrition Potsdam-Rehbruecke, Potsdam, Germany; 10 German Center for Diabetes Research (DZD), Munich-Neuherberg, 85764 München, Germany

**Keywords:** Depression, Leptin levels, Exercise, Body fat

## Abstract

**Background:**

Depression is a leading cause of disability worldwide and a significant contributor to the global burden of disease. Altered leptin levels are known to be associated with depressive symptoms, however discrepancies in the results of increased or decreased levels exist. Due to various limitations associated with commonly used antidepressant drugs, alternatives such as exercise therapy are gaining more importance. Therefore, the current study investigates whether depressed patients have higher leptin levels compared to healthy controls and if exercise is efficient to reduce these levels.

**Methods:**

Leptin levels of 105 participants with major depressive disorder (MDD; 45.7% female, age mean ± *SEM*: 39.1 ± 1.0) and 34 healthy controls (HC; 61.8% female, age mean ± *SEM*: 36.0 ± 2.0) were measured before and after a bicycle ergometer test. Additionally, the MDD group was separated into three groups: two endurance exercise intervention groups (EX) differing in their intensities, and a waiting list control group (WL). Leptin levels were measured pre and post a 12-week exercise intervention or the waiting period.

**Results:**

Baseline data showed no significant differences in leptin levels between the MDD and HC groups. As expected, correlation analyses displayed significant relations between leptin levels and body weight (HC: *r* = 0.474, *p* = 0.005; MDD: *r* = 0.198, *p* = 0.043) and even more with body fat content (HC: *r* = 0.755, *p* < 0.001; MDD: *r* = 0.675, *p* < 0.001). The acute effect of the bicycle ergometer test and the 12-week training intervention showed no significant changes in circulating leptin levels.

**Conclusion:**

Leptin levels were not altered in patients with major depression compared to healthy controls and exercise, both the acute response and after 12 weeks of endurance training, had no effect on the change in leptin levels.

**Trial registration:**

The study was registered at the German register for clinical studies (DRKS) and the International Clinical Trials Registry Platform of the World Health Organization https://trialsearch.who.int/Trial2.aspx?TrialID=DRKS00008869 on 28/07/2015.

**Supplementary Information:**

The online version contains supplementary material available at 10.1186/s12889-023-17362-4.

## Background

On a global scale, the World Health Organization (WHO) estimates that 5% of adults live with depression, a leading cause of disability worldwide and a significant contributor to the global burden of disease [1]. The number of patients with depression increased by 18.4% between 2005 and 2015, which is a significant increase compared to other mental illnesses [2]. In addition, the emergence of the COVID-19 pandemic has created an environment where many determinants of poor mental health are even exacerbated [3]. The persistent and ubiquitous nature of depression, coupled with its penchant to drive disability, morbidity, and mortality indicate the pressing need to develop innovative and broadly effective interventions [4]. The clinical prescription of antidepressant drugs remains the most common treatment for patients with depression, although typical antidepressants have various limitations in terms of therapeutic efficiency and onset time [5, 6]. Thus, other non-pharmacological and unconventional treatments, such as exercise, have been widely recommended due to their safety and non-toxic side effects [2]. Both the WHO [7] and the NICE guidelines [8] recommend implementing physical exercise in the standard treatment of depression. Based on systematic reviews and meta-analyses findings, exercise has an anti-depressant effect and even potential protective benefits [9–15]. The SPeED study (Sport/Exercise Therapy and Psychotherapy-evaluating treatment Effects in Depressive patients), a randomized controlled trial performed by our group, showed that a 12-week endurance exercise program with high intensity leads to an improvement in physical fitness compared to an endurance exercise program in low intensity and to a waiting list control group, while depressive symptoms improved in all groups [16, 17].

The multifactorial pathophysiology of depression, including disrupted neurogenesis, genetic susceptibility, and metabolic disturbance, complicates prediction of the disease and the individual response to treatment [18]. Recent studies suggested an association between leptin dysregulation and depression, possibly through brain neurogenesis pathways [19, 20]. As proposed in several basic studies, leptin has antidepressant effects and might be a potential therapeutic target for depression [21]. Leptin is one of the major hormones responsible for controlling energy balance and body weight by altering energy intake and energy expenditure [22, 23]. Leptin receptor (LepRb) deficiency leads to memory and cognitive impairments that are accompanied by alterations in hippocampal synaptic plasticity [24]. Acute administration of leptin has been reported to have antidepressant and anxiolytic effects in mice [25]. However, studies investigating the relationship of major depressive disorders (MDD) and leptin levels have yielded somewhat discrepant results. Initial studies reported lower leptin levels in depressive patients compared to healthy controls, which coincided with initial reports of leptin’s antidepressants effects in animal models [26–29]. Nevertheless, higher leptin levels in MDD have also been reported [30, 31].

Beyond that, several studies have analysed the effect of exercise on circulating leptin concentrations and it seems that the levels are only decreased by bouts of exercise with considerably high intensity and long duration [32]. But it is entirely unclear, whether acute or long-term exercise alters the circulating leptin levels in depressed patients and whether this is due to exercise-induced loss of body fat per se or via an independent mechanism. Therefore, in the current sub-project of the SPeED study, we aimed to investigate whether (i.) depressed patients have altered leptin levels compared to healthy controls, (ii.) acute and/or long-term endurance exercise influences leptin levels and (iii.) the intensity of the training determines the change in leptin.

## Methods

### Participants and eligibility criteria

105 out of 113 participants of the original SPeED study [16, 17] with a diagnosis of mild to moderate depression were included (MDD; 45.7% female, age mean ± *SEM*: 39.1 ± 1.0; range: 22–66 years) and compared to 34 healthy controls at baseline (t1) (HC; 61.8% female, age mean ± *SEM*: 36.0 ± 2.0; range: 21–60 years). For inclusion into the study, participants had to be diagnosed with mild or moderate depressive episode, an age between 18 and 65 years, and passing a sport medical examination. Past and present mental disorders were assessed in all participants by trained psychologists using the German version of the Structured Clinical Interview for DSM-IV TR (SCID) [33]. Participants were recruited between 07/23/2015 and 12/11/2019 and randomly assigned to one of three groups: high intensity exercise group (HEX), low intensity exercise group (LEX), and a waiting list control group (WL). Linke et al. [34] and Reljic et al. [35] both documented a higher dropout rate among participants in exercise groups when compared to those in the waiting list control groups. This led to a modified allocation ratio of 3:3:2 for the HEX, LEX, and WL group. An independent researcher, not affiliated with the study, generated the random allocation sequence using a computer program (MATLAB, The MathWorks Inc., Natick, USA). To maintain blinding procedures, participants were kept unaware of whether they were allocated to HEX or LEX, but informed about their allocation to either an exercise group or WL.

All participants, MDD and HC, passed a sport medical examination and were excluded with a current severe depressive episode with or without psychotic symptoms, the use of benzodiazepines or beta-blockers within the last 7 days, or tricyclic antidepressants and neuroleptic drugs with a dose of > 40% of the cumulated maximum recommended daily dose, a body mass index of > 35 or < 18, and more than 90 min of physical exercise per week (for detailed information on inclusion and exclusion criteria see [16] and supplementary material). The study protocol was approved by the local ethics committee of Charité Universitätsmedizin Berlin, Germany (No EA1/113/15) and the ethics committee of the Freie Universität Berlin, Germany (No 133/2016) and all methods were performed in accordance with the relevant guidelines and regulations. The study was registered at German register for clinical studies (DRKS) and the International Clinical Trials Registry Platform of the World Health Organization https://trialsearch.who.int/Trial2.aspx?TrialID=DRKS00008869 on 28/07/2015 and the study rationale, design, and methodological issues were reported in a previous publication [16]. Informed written consent was obtained from all participants.

### Procedure

Sport medical examination—To assess eligibility on the one hand and the effect of exercise on the other hand, all participants had to pass a sport medical examination, which included a graded bicycle ergometer test at baseline (t1) and after a 12-week intervention period (t2). The examinations were performed by a physician, a medical-technical assistant and a medical student, who were responsible for collecting anthropometric data, such as height, weight, body mass index, resting heart rate, and resting blood pressure, as well as conducting a medical anamnesis interview. During the bicycle test, continuous monitoring was ensured through electrocardiogram (ECG). Heart rate, blood pressure, and lactate levels were assessed before starting the test, at the end of each resistance level, and after completion. Blood samples were collected for platelet determination before and after bicycle test. The test started at 25 W with a graded increase in power of 25 W every 2 min until reaching the maximal physical exertion or the occurrence of general termination criteria according to the Borg scale [36]. See Kallies et al. [37] and Heinzel et al. [17] for further details of the physical fitness and physical activity assessment.

Intervention—The SPeED study was applied as a longitudinal design. To assess long-term training effects on leptin levels in depression, the MDD group received a 12-week endurance exercise program. The MDD participants were randomly assigned to three groups: two exercise intervention groups (EX) took part in a 12-week exercise program and were compared to a waiting list control group (WL). The EX groups received supervised endurance exercise training following the guidelines of the American Heart Association for endurance exercise [38], differing in their intensities, with 24 sessions in total and two sessions a week á 60 min. The supervised exercise program was conducted by two sport therapists with experience in the field of depression. Based on the data from the sports medical examination, the training intensities (Hftrain) of both EX groups were determined by the Karvonen formula using the heart rate reserve (Hfmax) and the resting heart rate (RP), HFtrain = (HFmax—RP) x factor + RP, [39]. In the HEX group, one session included a 20-min bicycle ergometer, continuing with 20-min of jogging or nordic walking and at the end with a 20-min aerobic workout. The participants chose on their own if they want to go jogging or nordic walking. No matter if they have chosen jogging or nordic walking they had to workout at approximately 55–85% of the individual maximum heart rate reserve in all parts. The LEX group received a similar exercise program, with the distinction of a 20-min bicycle ergometer, 20-min walking, and 20-min stretching and relaxation of about 20%-30% of the individual maximum heart rate reserve. Pulse watches and chest straps were used for the measurement of HF. During the exercise program, the heart rate and subjective feeling of exertion via Borg scale [36] was recorded after 3–5 min, 10–12 min and 18–20 min in each of the three parts of the program. Depending on HFmax and Borg scale the participants W/kg on the bicycle ergometer, the intensity during jogging or nordic walking, or in the aerobic part was adapted. Participants were able to choose on their own if they want to go jogging or nordic walking. Nevertheless, they had to do this in the percentage of maximum heart rate reserve. In both EX groups an attendance rate of 80% was ensured. All participants were asked not to start any additional regular and vigorous exercising outside the study protocol. Irrespective of their assigned group, all participants received the standard treatment offered Cognitive Behavioral Therapy (CBT) after 12 weeks of intervention. Particular attention was given to the WL group, with the aim to keep the waiting period for standard CBT treatment within or below the typical range of 3–6 months to prevent any potential disadvantages for study participants. It was also guaranteed that the same exercise program would be provided to the WL group after they had completed their participation in the study.

### Measures

Depression—The severity of depression was measured by the German version of the Beck Depression Inventory 2 (BDI-2) [40]. The BDI-2 consists of 21 items rated on a 4-point Likert scale ranging from 0 (non-existent) to 3 (strong expression). With a total score ranging from 0–63, the BDI-2 is a self-report instrument that classifies the severity of depression into the following categories according to the BDI-2 manual of Beck et al. (1996): a score of 0–13 indicates minimal depression, 14–19 mild depression, 20–28 moderate depression, and 29–63 severe depression. With a Cronbach´s α = 0.81 the reliability in the present study showed good internal consistency.

Blood samples—Serum levels of leptin were measured prior to and after the bicycle ergometer tests at baseline (t1) in all participants and after the 12-week intervention period (t2) in the exercise groups and waiting list control group. To quantify leptin, venous blood samples were drawn from the antebrachial vein after a rest period of at least 20 min (pre-exercise) and post-exercise samples were collected within 5 min after completion of the bicycle ergometer test. Blood was allowed to clot for 60 min at room temperature before centrifugation at 1,300 × g and 20 °C for 10 min. The resulting serum fraction was aliquoted into microliter tubes and stored at -30 °C for further analysis. For leptin measurements, an enzyme-linked immunosorbent assay (ELISA) method was performed using the Human Leptin Quantikine ELISA Kit (DLP00) from Biotechne (R&D Systems Inc., a Bio-Techne Brand, Minneapolis, Minnesota, United States) with a detection range of 15.6—1,000 pg/ml. All samples were analysed in duplicate at the same time after completion of the study.

### Statistical analysis

All data were processed and statistically analyzed using SPSS 28 (IBM Corp. Released 2021. IBM SPSS Statistics for Windows, Version 28.0. Armonk, NY: IBM Corp). Continuous data are summarized as mean and SEM. The Shapiro–Wilk test was used to test for the normal distribution of variables. For correlation analysis, Pearson´s correlation test was used. Paired sample t-test were applied to compare baseline characteristics between patients with MDD and HC. Repeated measures analyses of variances (rmANOVA) were done to compare the effect of the acute exercise bicycle ergometer test on circulating leptin mean levels and whether there were statistically significant differences between MDD and HC. Further rmANOVA were done to evaluate the change in mean leptin levels and the influence of group membership pre and post the intervention period. Differences were considered significant at *p* < 0.05 for all tests. Sensitivity analysis was performed by using analysis of covariance (ANCOVA) model to test phenotypic traits as covariates, like body fat in exercise induced leptin alterations and the effect of the time of inclusion concerning the recruitment period and storage of plasma that may have an influence on leptin levels. For better comprehension of the mechanisms underlying the effect of training on leptin levels and change in depression severity, regression analysis was performed regarding the prediction of attendance-rate in the EX group and a possible prediction of leptin levels on the change in BDI-2 scores in participants with MDD. The change in BDI2 scores was calculated using: (BDI-2 sumscore t2—BDI-2 sumscore t1).

## Results

The present sub-project of the SPeED study aimed to investigate the difference in circulating leptin levels between depressed patients and healthy controls and to examine the impact of acute and long-term endurance exercise on leptin levels.

### Baseline characteristics

**Fig. 1:**
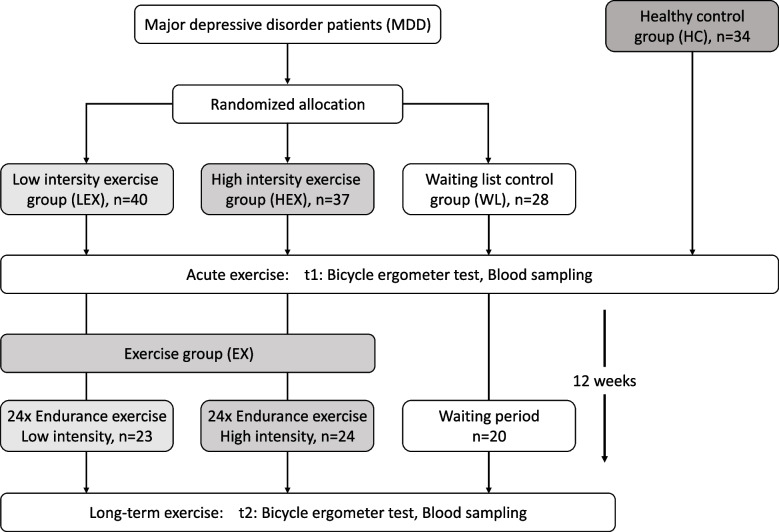
Study design

**Fig. 2:**
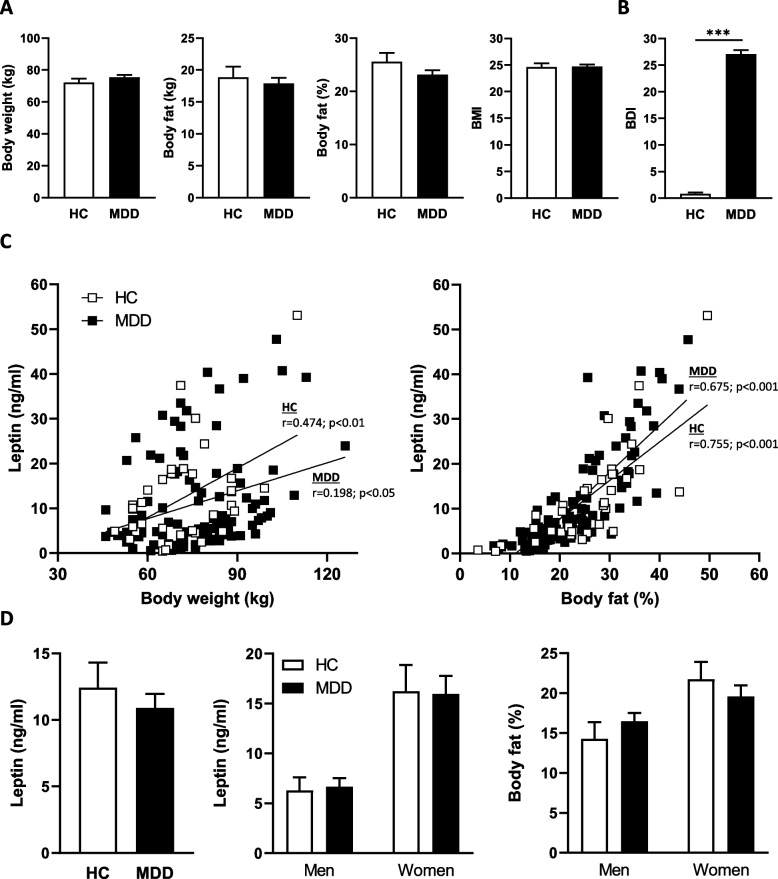
Baseline data of study participants. Comparison of (**A**) body weight, body fat content, percent body fat, and body mass index (BMI) in patients with major depressive disorder (MDD, *n* = 105) and healthy controls (HC, *n* = 34). **B** Beck Depression Inventory-2 (BDI-2) between MDD and HC at baseline. **C** Bivariate correlation analysis between body weight and serum leptin levels (left panel) and percent body fat and serum leptin levels (right panel) in MDD and HC. **D** Serum leptin levels in patients with MDD (left panel) compared to controls (HC) as well as leptin values (middle panel) and percent body fat (right panel) depending on sex and depression status. Data are shown as mean ± SEM. *P < 0.05; ***P < 0.00001

Within the presented sub-project of the SPeED study, 105 patients with major depressive disorder (MDD) were compared with 34 healthy controls (HC) as reported in the flow chart in Fig. 1. At baseline, the HC and MDD group had comparable anthropometric characteristics (Fig. 2A) including the phenotypic traits body weight (72.2 ± 2.4 vs. 75.4 ± 1.6; *p* = 0.296), body fat (18.9 ± 0.9 vs. 17.9 ± 0.8; *p* = 0.589), percent body fat (25.6 ± 1.7 vs. 23.1 ± 0.8; *p* = 0.163) as well as a similar body mass index (BMI) (24.6 ± 0.7 vs. 24.7 ± 0.4; *p* = 0.929). Depressive symptoms (BDI-2) were significantly higher in the MDD group (0.82 ± 0.27 vs. 27.08 ± 0.77; *p* < 0.001) (Fig. 2B).

In the current study, bivariate correlation analyses also showed a positive correlation between leptin levels and body weight (HC: *r* = 0.474, *p* = 0.005; MDD: *r* = 0.198, *p* = 0.043). Given the numerical difference in the magnitude of the association between depressed patients and healthy controls, we calculated a z-test to determine whether the association between body weight and leptin levels was attenuated in depressed patients, but this difference proved not be significant (z = 1.46, *p* = 0.14). For the percentage of body fat, we found an even stronger correlation of equal magnitude in both groups, HC and MDD (HC: *r* = 0.755, *p* < 0.001; MDD: *r* = 0.675, *p* < 0.001) (Fig. 2C). Similarly, no difference in leptin levels was detected between patients with depression and healthy controls (HC: 12.43 ± 1.89 vs. MDD: 10.91 ± 1.05, *p* = 0.479) (Fig. 2D, left panel). At baseline, leptin values revealed a significant correlation in the MDD group (*r* = 0.226, *p* = 0.021), but the mean values were nevertheless lower than in healthy controls.

The subdivision of the individual groups according to sex showed, as expected, significantly higher values for women than for men, but again independent of the depression status (Fig. 2D). In conclusion, circulating leptin levels were not altered in patients with major depression compared to healthy controls.

### Effects of acute exercise

**Fig. 3:**
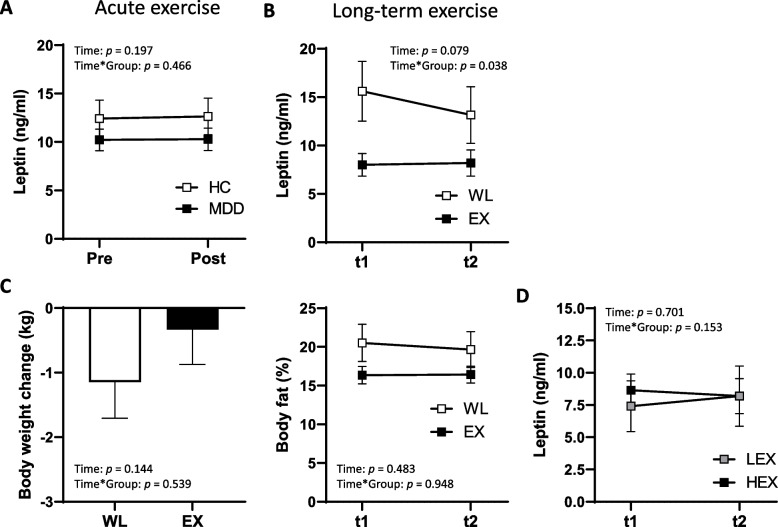
Change of leptin levels after acute and long-term exercise. **A** Serum leptin levels in patients with depression (MDD) compared to controls (HC) before (Pre) and after (Post) bicycle ergometer test. **B** Analysis of circulating leptin levels in patients with major depression before (t1) and after (t2) a 12-week exercise intervention (EX, *n* = 77) in comparison to a waiting list control group (WL, *n* = 28). **C** Change in body weight and percent body fat before and after long-term exercise in the EX versus WL group. **D** Subdivision of the exercise group depending on the training intensity (low intensity, LEX, *n* = 40; high intensity, HEX, *n* = 37) before (t1) and after (t2) long-term exercise intervention and analysis of circulating leptin values. Data are shown as mean ± SEM

To test whether leptin levels change after a single bout of exercise and were dependent on the depression status, all participants performed a bicycle ergometer test. As shown in Fig. 3A, the change of leptin levels (Time: F(1,137) = 1.678, *p* = 0.197, partial η^2^ = 0.012, Time*Group: F(1,137) = 0.534, *p* = 0.466, partial η^2^ = 0.004) was not significantly different between pre- and post- acute exercise and not different between MDD patients and HC, showing that the bicycle ergometer test had no effect on the change in leptin levels, nor that the response was altered in depression.

### Long-term effects of endurance exercise

All MDD patients were randomly allocated to one of three groups; a low-intensity (LEX) or a high-intensity (HEX) endurance exercise group (EX) or a waiting list control group (WL) (Fig. 1). Overall, the comparison of EX and WL revealed a marginally significant main and significant interaction effect (Time: F(1,65) = 3.176, *p* = 0.079, partial η^2^ = 0.047; Time*Group: F(1,65) = 4.474, *p* = 0.038, partial η^2^ = 0.064). In contrast to the exercise group with similar leptin levels pre- and post-exercise (pre: 8.01 ± 8.02; post: 8.19 ± 9.25) intervention, leptin levels of the waiting group (pre: 15.00 ± 13.37; post: 12.91 ± 12.42), which were higher at baseline, decreased after 12 weeks (Fig. 3B, time: F(1,65) = 3.176, *p* = 0.079, partial η^2^ = 0.047, Time*Group: F(1,65) = 4.474, *p* = 0.038, partial η^2^ = 0.064.). There was no main effect nor an interaction effect of exercise on body weight (Time: F(1,65) = 0.381, *p* = 0.144, partial η^2^ = 0.033; Group*Time: F(1,65) = 0.381, *p* = 0.539, partial η^2^ = 0.006) and percent of body fat (Time: F(1,65) = 0.497, *p* = 0.483, partial η^2^ = 0.008; Group*Time: F(1,65) = 0.004, *p* = 0.948, partial η^2^ = 0.000) from pre to post intervention (Fig. 3C), but body fat content was higher in the WL pre (t1) and post (t2) exercise than in the EX groups (Fig. 3C). Furthermore, the EX group had a lower percentage of body fat compared to the WL at baseline (t1, Fig. 3C) and this initial difference in body fat between WL and EX groups may explain the higher leptin levels in the WL at baseline as well as the subsequent stronger decrease in leptin levels over time. An independent sample t test revealed a statistically significant difference in percent of body fat at baseline between EX and WL, t(28.05) = -2.155, *p* = 0.040*. Since a considerable interaction effect of group membership on the leptin levels was observed, a possible impact of percent of body fat on the change in leptin levels was investigated by using an ANCOVA, taking leptin levels before and after the intervention as dependent variables, the group’s membership as the fixed factor and body fat as a covariate. The ANCOVA revealed that the interaction of the change in leptin levels and group membership was no longer significant when taking body fat as covariate, Time: F(1,64) = 0.044, *p* = 0.834; partial η^2^ = 0.001; Time*Body fat: F(1,64) = 0.164, *p* = 0.687 partial η^2^ = 0.003; Time*Group: F(1,64) = 3.587, *p* = 0.063, partial η^2^ = 0.053.

In terms of training intensity, results of the rmANOVA considering leptin levels showed no significant changes indicating that there was no overall effect of exercise on leptin levels and this is independent of the exercise intensity (Time: F(1,45) = 0.150, *p* = 0.701, partial η^2^ = 0.003; Time*Group: F(1,45) = 2.117, *p* = 0.153, partial η^2^ = 0.045). However, the concentration of leptin levels decreased numerically in the HEX group, while the levels increased in the LEX group (Fig. 3D). When considering the time of inclusion into the study as a possible impact on the change in leptin scores, due to the recruiting period and storage time of blood samples an ANCOVA showed no significant results (see supplementary material for further information).

Regarding the effect of the exercise intervention on depressive symptoms, a significant reduction in BDI-2 scores was found, but no interaction between the exercise groups and WL (Time: F(1,65) = 21.330, *p* < 0.001, partial η^2^ = 0.247; Time*Group: F(1,65) = 0.851, *p* = 0.360, partial η^2^ = 0.013). Leptin levels at baseline also did not predict the change in BDI-2 scores (see supplementary material).

## Discussion

Studies investigating the relationship between major depressive disorder (MDD) and leptin levels revealed conflicting results, and it is so far unclear whether endurance training alters leptin levels. Therefore, within the sub-project of the SPeED study, circulating leptin levels of patients with depression were compared with healthy controls before and after an acute exercise bout and long-term exercise intervention. Overall, leptin levels were not altered in patients with major depression compared to healthy controls, and exercise, both acutely and after 12-week endurance training, had no effect on the change in leptin levels. The training intensity showed no significant differences in the effect of exercise on serum leptin.

The role of leptin in regulating energy homeostasis, in which it acts as a negative feedback adiposity signal in specific hypothalamic nuclei, is well described. Within the arcuate nucleus of the hypothalamus (ARC), leptin acts on leptin receptors (LepRb) to inhibit neurons that express the orexigenic (appetite-stimulating) neuropeptide agouti-related peptide (AgRP), while simultaneously stimulating nearby neurons that express the anorexigenic (appetite-suppressing) neuropeptide proopiomelanocortin (POMC). Both of these actions work together to reduce food intake [41, 42]. Besides that, there is growing experimental results indicating that leptin also plays a significant role in other areas of the central nervous system (CNS) and is associated with several pathological and physiological mechanisms of neurological diseases, including neurodegenerative diseases and mood disorders [43, 44]. Leptin influences neurotransmitters such as dopamine [45] and impacts gray matter plasticity [46]. It was found that neurological diseases occurred alongside leptin level alterations, suggesting that leptin might be a critical modulator of these diseases, and studying the specific relationship is of significance [47]. Available information about the role of leptin signaling in human depression is limited and controversial. Similar to our findings, the first study analyzing plasma leptin concentrations in depressed patients reported no difference between depressed patients and healthy controls, despite a weight loss in the patients examined [48]. Nevertheless, several clinical studies have found lower serum leptin levels in individuals with MDD compared to healthy controls [26, 28], whereas other data in women with MDD revealed significantly increased plasma leptin levels [30, 31, 49]. Similarly, some studies reported that leptin concentrations are variously increased [30, 50] or not changed by antidepressant treatment [50, 51]. Lastly, some studies suggest that leptin may be a biomarker of risk for de-novo depression [52, 53]. One possible explanation for the seemingly contradictory data may be that leptin levels are influenced by certain factors such as age, sex, body mass status, medication history, and comorbidity with other disorders [21]. Another interpretation is that leptin insufficiency may only occur in a subpopulation of depressed patients [54]. The systematic review and meta-analysis by Carvalho et al. underscore that relevant moderators/confounders (e.g., BMI, depression severity, and type of assay) should be controlled for when considering the role of leptin as putative MDD diagnostic biomarker [53].

In the current study, as expected, serum leptin levels were positively associated with body weight and even more with body fat content and, moreover, differed depending on sex. The difference in leptin concentrations between men and women is due to body fat, BMI, and the action of sex hormones [55–57]. Luukkaa et al. showed an inverse relationship between testosterone and leptin concentrations. But nevertheless, also the subdivision into women and men revealed no difference regarding leptin levels and the status of depression in the current study [58].

While it stands to reason that exercise and physical activity affect energy balance, the effects of endurance exercise on plasma leptin are somewhat inconsistent. Long-term exercise training resulted in decreased circulating leptin levels, but these reductions were largely driven by improvements in body composition [59, 60]. Although a decrease in percent body fat appears to be one of the most significant factors associated with a decrease in leptin levels, several chronic exercise interventions also reported a decrease regardless of body weight loss [61–63].

Studies examining the leptin response to single bouts of exercise have produced equivocal results. In a study with unconditioned male volunteers, leptin concentrations declined following acute exercise reaching nadir values 30–120 min after exercise [64] and similar results were observed in trained rowers, where leptin concentration decreased immediately after and 30 min after maximal rowing exercise (30 min) [65]. No alterations in circulating leptin were reported in sedentary adult men and women after completion of an acute exercise test on cycle ergometer [59] and short-term exercise in obese females, walking at 60–80% of the heart rate maximum (HRM) for 45 min also did not alter leptin concentrations [66]. With regard to long-term exercise, in female subjects a decline in serum leptin levels were detected after a 12-wk training intervention [62] and was similar to results obtained by Ishii et al., where lower levels were reported in type 2 diabetic subjects after 6 weeks of moderate-intensity aerobic exercise training [63]. Bouassida et al., reviewed the findings of different studies considering the effects of acute and chronic exercise on leptin levels and it appears that lowered leptin concentrations were observed after long-term exercise (> 60 min) that stimulates FFA release, or after exercise that generates an energy expenditure higher than 800 kcal [67]. Controversially, the results of the current study, with a 12-week exercise intervention, do not show a reduction in leptin levels after acute and long-term exercise. Moreover, in terms of training intensity, no significant differences could be found, although there is a numerically tendency showing that leptin values decreased after a high intensity exercise training while levels increased after a low intensity exercise training. It is possible that putative leptin resistance is positively affected by the training without changing circulating leptin concentrations. An improved leptin sensitivity may contribute to a reduction in depression. Compared to similar levels pre and post intervention in the exercise group, serum leptin decreased in the waiting list control group. However, this difference can most likely be attributed to a higher body fat percentage in the control group before the intervention and a tendency towards reduced body fat and weight after 12 weeks.

## Conclusions

In summary, and referring to the initial research questions, the sub-project of the SPeED study showed that (i.) depressed patients had similar leptin levels compared to healthy controls, (ii.) no change in circulating leptin levels after an acute and endurance exercise intervention, and (iii.) the intensity of exercise had no impact on serum leptin in depressed patients.

### Supplementary Information

**Additional file 1:** Supplementary information. **Text 1.** Exclusion criteria. **Text 2.** Further statistical calculations.

## Data Availability

The datasets used and/or analysed in the present study are available from Stephan Heinzel (heinzel@zedat.fu-berlin.de) upon reasonable request.

## Abbreviations

ANCOVA: Analysis of covariance
BDI-2: Beck Depression Inventory 2
BMI: Body mass index
CNS: Central nervous system
ELISA: Enzyme-linked immunosorbent assay
HC: Healthy controls
HEX: High intensity exercise group
LepRb: Leptin receptor
LEX: Low intensity exercise group
MDD: Major depressive disorder
NICE: National Institute for Health and Care Excellence
rmANOVA: Repeated measures analysis of variance
SEM: Standard error of the mean
SPeED: Sport/Exercise Therapy and Psychotherapy-evaluating treatment Effects in Depressive patients
WHO: World Health Organization
WL: Waiting list control group
