# Supplementary material for: Effect of acute and long-term exercise on leptin levels in depressed outpatients
Source: BMC Public Health. 2023 Dec 14;23:2509. doi: 10.1186/s12889-023-17362-4 (PMC10722655; doi:10.1186/s12889-023-17362-4)
Supplement: Supplementary file 1 — Additional file 1: Supplementary information. Text 1. Exclusion criteria. Text 2. Further statistical calculations. [file 12889_2023_17362_MOESM1_ESM.docx]

Supplementary information

**Effect of acute and long-term exercise on leptin levels in depressed outpatients**

Darlene Heinen^a,b^, Andreas Heissel^c,d^, Stephan Heinzel^e^, Thomas Fydrich^f^, Andreas Ströhle^g^, Michael Rapp^c^, Heike Vogel^b,h,i^

**Text 1.** Exclusion criteria

**Text 2.** Further statistical calculations

**Supplementary information Text 1. Exclusion criteria**

The following exclusion criteria were applied to all

patients:

- current severe depressive episode with or without psychotic symptoms
- current borderline or antisocial personality disorder
- current suicidality
- lifetime schizophrenia spectrum disorder
- delusional disorder
- bipolar disorder
- current alcohol or drug addiction
- current severe neurological diseases of the central nervous system
- severe chronic obstructive pulmonary disease
- coronary heart disease and heart failure
- body mass index of > 35 or < 18
- insufficient language abilities in German
- uncorrectable visual or hearing impairments
- magnetic resonance imaging unsuitability
- use of benzodiazepines or beta-blockers within the last 7 days
- tricyclic antidepressants and neuroleptic drugs with a dose of > 40% of the cumulated maximum recommended daily dose
- more than 2 X 45-min physical exercise per week.

**Text 2.** **Further statistical calculations**

ANCOVA:

ANCOVA analysis was done for calculating the effect of the intervention on the change scores of leptin levels after adjusting for the time of sports medical examination.

Time of sport medical examination:

The ANCOVA for analyzing the different effects of exercise intensity (HEX and LEX) and WL on the change score in leptin levels after adjusting for the time of the sport medical examination showed no significant results (F(1,61)= 0.174, p= 0.678, partial η²= 0.010). Also no effect was found when comparing EX with WL (F(1,63)=2.119, p=0.150, partial η²= 0.033).”

Regression analysis:

Regression analysis was done to prove the prediction of leptin level on the change in BDI-2 scores. Leptin level cannot predict the change in BDI-2 scores, *p* = 0.601. With R² of 0.004 the model has no fit.”

The regression analysis of the prediction of the attendance-rate, concerning the exercise intervention on change scores in leptin levels showed no significant results, *p* = 0.868, R² = 0.001.
